# 3D bioprinting of dECM-incorporated hepatocyte spheroid for simultaneous promotion of cell-cell and -ECM interactions

**DOI:** 10.3389/fbioe.2023.1305023

**Published:** 2023-11-13

**Authors:** Min Kyeong Kim, Wonwoo Jeong, Seunggyu Jeon, Hyun-Wook Kang

**Affiliations:** ^1^ Department of Biomedical Engineering, Ulsan National Institute of Science and Technology, Ulsan, Republic of Korea; ^2^ Center for Scientific Instrumentation, Korea Basic Science Institute, Chungbuk, Republic of Korea; ^3^ Wake Forest Institute for Regenerative Medicine, Wake Forest University School of Medicine, Winston-Salem, NC, United States

**Keywords:** liver tissue engineering, dECM-incorporated hepatocyte spheroid, cell-ECM interaction, decellularization, 3D bioprinting

## Abstract

The cell spheroid technology, which greatly enhances cell-cell interactions, has gained significant attention in the development of *in vitro* liver models. However, existing cell spheroid technologies still have limitations in improving hepatocyte-extracellular matrix (ECM) interaction, which have a significant impact on hepatic function. In this study, we have developed a novel bioprinting technology for decellularized ECM (dECM)-incorporated hepatocyte spheroids that could enhance both cell-cell and -ECM interactions simultaneously. To provide a biomimetic environment, a porcine liver dECM-based cell bio-ink was developed, and a spheroid printing process using this bio-ink was established. As a result, we precisely printed the dECM-incorporated hepatocyte spheroids with a diameter of approximately 160–220 μm using primary mouse hepatocyte (PMHs). The dECM materials were uniformly distributed within the bio-printed spheroids, and even after more than 2 weeks of culture, the spheroids maintained their spherical shape and high viability. The incorporation of dECM also significantly improved the hepatic function of hepatocyte spheroids. Compared to hepatocyte-only spheroids, dECM-incorporated hepatocyte spheroids showed approximately 4.3- and 2.5-fold increased levels of albumin and urea secretion, respectively, and a 2.0-fold increase in CYP enzyme activity. These characteristics were also reflected in the hepatic gene expression levels of *ALB*, *HNF4A*, *CPS1*, and others. Furthermore, the dECM-incorporated hepatocyte spheroids exhibited up to a 1.8-fold enhanced drug responsiveness to representative hepatotoxic drugs such as acetaminophen, celecoxib, and amiodarone. Based on these results, it can be concluded that the dECM-incorporated spheroid printing technology has great potential for the development of highly functional *in vitro* liver tissue models for drug toxicity assessment.

## 1 Introduction

The liver is the main organ responsible for various types of metabolisms and the detoxification of external toxins and chemical compounds within the body. In particular, drug-induced liver injury is one of the main causes of drug withdrawals because drug metabolism primarily occurs in the liver (Kaplowitz, 2005). Therefore, assessing liver toxicity is an essential process in drug development. In this regard, several *in vitro* liver models for drug toxicity testing have been developed (Khetani and Bhatia, 2008; Nguyen et al., 2016; Bircsak et al., 2021; Lee et al., 2021). Primary hepatocytes are widely used in liver tissue model development due to their higher functionality compared to stem cells and cell lines, allowing for a more precise evaluation of liver toxicity. However, the decreased viability and functionality of primary hepatocytes after isolation made significant challenges when applied as *in vitro* liver models (Guillouzo, 1998; Zeilinger et al., 2016). To overcome the limitations, 3D spheroid culture techniques that greatly increase cell-cell interactions have been actively applied in the study of liver tissue model (Bell et al., 2016; Vorrink et al., 2017; Zhang et al., 2023). 3D spheroid culture not only enhanced the expression levels of proteins related to drug absorption, distribution, metabolism, and excretion functions of primary hepatocytes but also significantly increased sensitivity to hepatotoxic compounds (Bell et al., 2018). Although these 3D spheroid culture techniques effectively improved cell-cell interactions, they have limitations in enhancing cell-extracellular matrix (ECM) interactions.

Within liver tissue, hepatocytes are polarized with the apical and basal surfaces. The basal surfaces of hepatocytes interact with ECM components in the space of Disse, and these interactions are important for maintaining hepatocyte polarity and functionality (Dunn et al., 1989; Treyer and Musch, 2013). Therefore, both cell-cell and -ECM interactions are essential factors to consider in the development of liver tissue structures (Vinken et al., 2006). In this regard, research aimed at enhancing hepatocyte functionality using various biomaterials has been actively conducted (MacPherson et al., 2021). Additionally, the hepatic functionality of hepatocyte 3D spheroid form can also be improved through the application of various functional biomaterials (Kim et al., 2010; Skardal et al., 2015). Deng et al. (Deng et al., 2021) introduced a liver tissue model which hepatocyte spheroids were encapsulated in an alginate hydrogel containing various ECM materials such as collagen and laminin. They demonstrated that the 3D spheroid encapsulated in ECM materials significantly helped maintenance of liver cell polarity. In addition, they showed that these ECM materials were suitable for preserving the spheroid morphology and enhancing albumin and urea secretion levels. However, the traditional spheroid culture method encapsulated in the ECM scaffold was hard to fully consider the existence of ECM interaction inside the spheroid (Yamada et al., 2015). Therefore, the development of 3D hepatocyte spheroid technology that can simultaneously enhance both cell-cell and -ECM interactions is needed.

In this regard, recent research has introduced techniques that allowed the incorporation of ECM materials within hepatocyte spheroids. Tao et al. (Tao et al., 2020) introduced a technique for creating Matrigel or collagen-loaded hepatic cell spheroids. They demonstrated that the production of ECM-loaded spheroids significantly enhanced the expression of cell polarity-associated proteins. Additionally, techniques for spheroid formation using ECM microparticles or microfibers had also been introduced (Ajoudanian et al., 2019; Heydari et al., 2021; Utoh et al., 2021). Yamada et al. (Yamada et al., 2015) introduced a method for creating composite spheroids using collagen microparticles with primary hepatocytes and showed that this approach could significantly enhance hepatocyte-specific gene expression levels. Thus, several techniques have been introduced to enhance the function of hepatocyte spheroids using biomaterials. However, these techniques used homogeneous materials like collagen, and there have been limitations in achieving a biochemical similarity with the complex ECM composition of *in vivo* liver tissue. Furthermore, the technologies have heavily relied on manual processes, which induce failures in spheroid self-assembly and decrease precision and reproducibility.

To address this issue, herein, we developed a 3D printing technology for decellularized ECM (dECM)-incorporated hepatocyte spheroids, allowing for the simultaneous enhancement of cell-cell and -ECM interactions. The developed printing technology used dECM hydrogel to evenly distribute tissue-specific ECM between cells for effectively enhancing cell-ECM interaction. To achieve this, a liver dECM-based cell bio-ink with a complex biochemical composition similar to actual liver tissue was prepared through the decellularization process and was used to fabricate ECM-incorporated hepatocyte spheroids. Through histological assays, we analyzed the ECM distribution within the spheroids according to the characteristics of bio-inks and optimized them accordingly. Subsequently, we examined the hepatic functionalities of the printed spheroids through albumin and urea secretion and CYP enzyme activity analysis. Finally, through toxicity evaluations using various drugs, we validated the feasibility of the developed dECM-incorporated hepatocyte spheroids as a liver toxicity test platform for drug development.

## 2 Materials and methods

### 2.1 Preparation and analysis of liver dECM

The porcine liver-derived dECM was fabricated using the protocol of previous study (Jeong et al., 2021). In brief, porcine liver obtained from a slaughterhouse was chopped and gently washed with distilled water. To remove cell components, the washed tissue was immersed in a solution containing 1% v/v Triton X-100 (Samchun Chemical, Republic of Korea) and 0.1% v/v ammonia solution (Samchun Chemical) for 48 h at 4 °C with agitation. The remaining detergent and cellular components were removed using distilled water for another 48 h at 4°C.

For evaluation of the dECM, histological and biochemical analyses were conducted. For histological analysis, native and decellularized liver tissues were fixed in a 4% paraformaldehyde solution (Samchun Chemical) at 4°C for overnight, followed by washing with distilled water. After tissue processing and tissue embedding, samples were sectioned at 5 μm thickness using a microtome (Leica, Germany) and then deparaffinized. The tissue slides were stained with hematoxylin and eosin (H&E, Abcam, UK). DNA content was quantified through genomic DNA extraction. In brief, the native and decellularized tissue samples were lysed using a Tris-EDTA buffer (Bioneer, Republic of Korea) with 1% v/v sodium dodecyl sulfate (Bioneer) and 1.0 mg/mL proteinase K (Bioneer). 5 M NaCl (Samchun Chemical) was treated to precipitate genomic DNA. The precipitated genomic DNA was dissolved in distilled water, and the DNA concentration was measured using a NanoDrop (Thermo Fisher Scientific, United States). The glycosaminoglycans (GAGs) and collagen concentration in native and decellularized tissues were measured using the Blyscan GAGs assay kit (Biocolor Life Sciences, UK) and QuickZime Total Collagen assay kit (QuickZime Bioscience, Netherlands) respectively, following the manufacturer’s instructions.

The prepared dECM powder (1 g) was digested using 0.01 N HCl solution (Sigma) with pepsin (100 mg, porcine pepsin, Sigma) for 48 h at 18 °C. After the digestion process, 10% v/v of 10X phosphate buffered saline (PBS, Sigma) was added and neutralized using a 0.5 N NaOH solution (Sigma) to make the digested dECM solution. The neutralization process was carried out on ice to prevent crosslinking of the dECM pre-gel.

### 2.2 Bio-inks preparation and viscosity analysis

The matrix ink and dECM-based cell bio-ink were prepared for dECM-incorporated spheroid printing. The matrix ink was composed of hyaluronic acid (HA), alginate, and gelatin. In detail, HA (3 mg/mL, Sigma) was dissolved in Minimum Essential Medium (MEM, Corning, United States) overnight at 37°C with gentle rotation. Then, alginate (15 mg/mL, Sigma) and gelatin (22.5 mg/mL, Sigma) were dissolved for 2 h at 37°C with gentle rotation. The matrix ink was sterilized using a 0.45 μm filter and store at −80 °C until used.

The dECM-based cell bio-ink was a mixture of digested dECM solution and sacrificial bio-ink. The sacrificial bio-ink was consisting of gelatin, HA, and CaCl_2_. In detail, HA (6 mg/mL) was dissolved in MEM overnight at 37°C with gentle rotation. Then, CaCl_2_ (80 mM, JUNSEI, Japan) and gelatin (25–65 mg/mL) were dissolved in the HA solution for 2 h at 37°C with gentle rotation, followed by sterilization using a 0.45 μm filter. The sterilized sacrificial bio-ink was store at −80 °C until used. An equal volume ratio of the digested dECM solution and the sacrificial bio-ink were homogeneously mixed create the dECM-based cell bio-ink. Finally, dECM-based cell bio-inks consisting of HA (3 mg/mL), CaCl_2_ (40 mM), gelatin (12.5–32.5 mg/mL), and dECM (0%–1.0% w/v) were prepared. For collagen incorporated spheroid printing, collagen (Atellocollagen type I, Darim Tissen, Republic of Korea) hydrogel was prepared using the same dECM digestion process and used instead of the dECM.

The viscosity of the matrix ink and dECM-based cell bio-ink with various gelatin and dECM concentrations was analyzed. To measure the rheological properties of each bio-ink, a shear sweep analysis with a shear rate of 0.1–100 S^−1^ was conducted using a HAAKE MARS III Rheometer (Thermo Fisher Scientific, United States) at 18°C.

### 2.3 Cell isolation and cell-laden bio-ink preparation

Primary mouse hepatocytes (PMHs) were utilized for the dECM-incorporated spheroid printing. PMHs were isolated from 8 weeks old C57BL/6J male mice using a two-step collagenase perfusion procedure (Seglen, 1976). The viability of the isolated PMHs was confirmed using trypan blue exclusion test, and cells with more than 90% viability were used. Prior to bio-printing, the 5 × 10^7^ cells/ml of hepatocytes were homogeneously mixed with cell bio-inks and loaded into a 1-mL syringe. All isolation procedures were approved by the Institutional Animal Care and Use Committee of UNIST (IACUC protocol number: UNISTIACUC-20–50).

### 2.4 dECM-incorporated spheroid printing process

**FIGURE 1:**
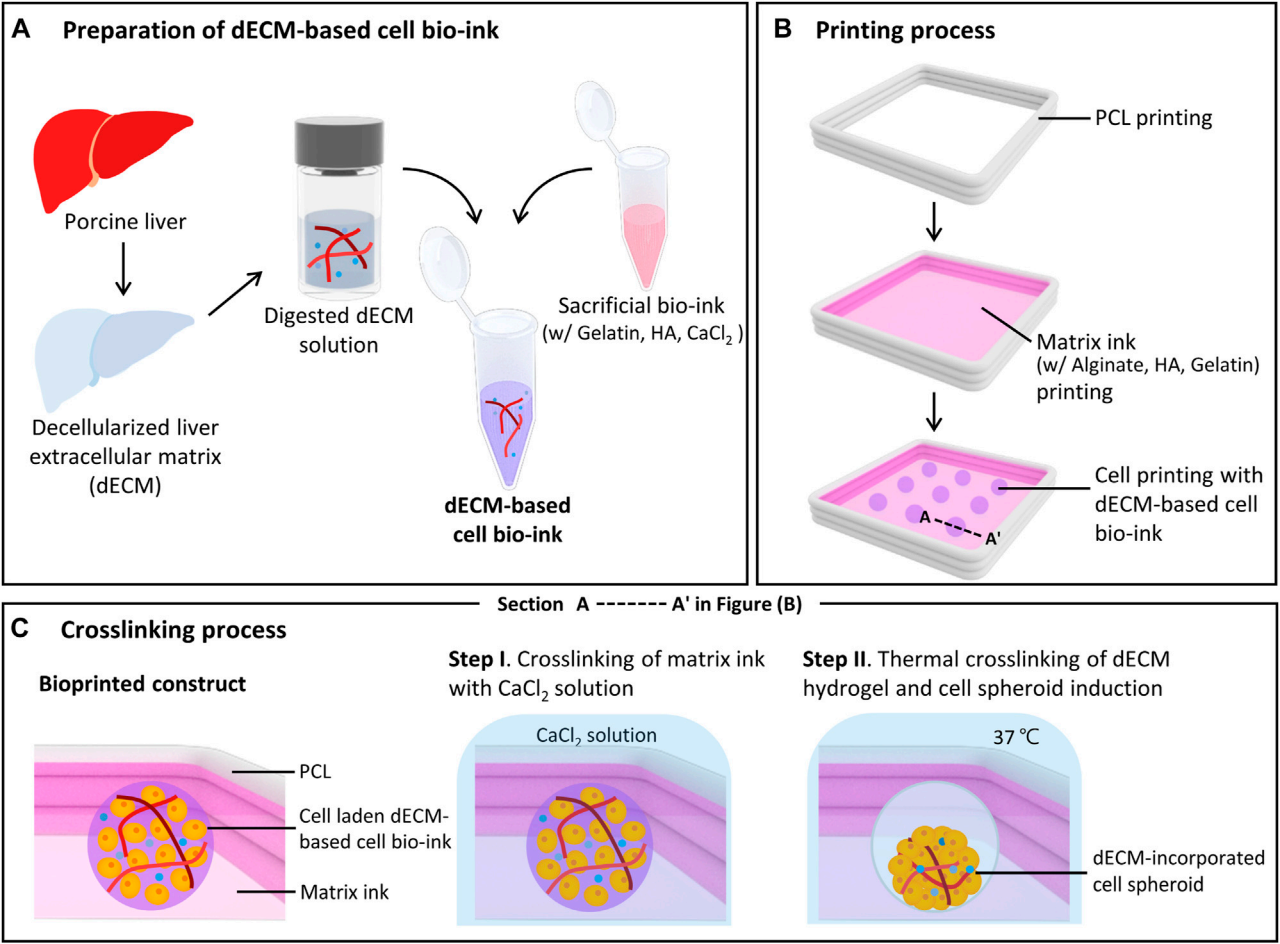
Bioprinting process of decellularized extracellular matrix (dECM)-incorporated cell spheroids. Schematic illustrations of dECM-based cell bio-ink preparation **(A)**, spheroid printing process **(B)**, and detailed crosslinking process **(C)**. Figure **(C)** shows a cross-sectional view of the printed construct in figure **(B)**. (HA: Hyaluronic acid, PCL: Polycaprolactone).

The dECM-incorporated spheroid was printed using the custom designed bioprinting system. This bioprinting system consists of a three-axis stage, multi-cartridge dispensing modules, and an enclosure to maintain humidity and temperature. To prepare dECM-based cell bio-ink, digested porcine liver dECM solution and sacrificial bio-ink were homogeneously mixed (Figure 1A). Figure 1B shows the printing process of dECM-incorporated cell spheroids. Firstly, a frame was printed with polycaprolactone (PCL) using a 200 μm metal nozzle at 90 °C. Then, the matrix ink was printed to fill the PCL frame with a dispensing rate of 0.221 μL/s and a printing speed of 35 mm/min, using a 250 μm metal nozzle. Next, the prepared dECM-based cell bio-ink was printed to produce cell spheroid with 0.0069 μL/s dispensing rate and 3 s dispensing time using a 120 μm needle nozzle. Based on the extrusion volume of dECM-based cell bio-inks into the printed matrix ink and the dispensing time, a total volume of 0.0207 μL was printed for each spheroid, with a corresponding dECM amount of 0.1035 μg per spheroid for the 0.5% w/v dECM group. Figure 1C depicts the section view of A-A’ in Figure 1B during the crosslinking process. The 40 mM CaCl_2_ selectively crosslinked the alginate within the matrix ink for 5 min, and then the structures were incubated at 37 °C for 25 min. During the incubation, non-crosslinked and soluble materials such as HA and gelatin were dissolved, which created circular cavities in the matrix ink. At the same time, dECM pre-gel in the cell bio-ink was thermally crosslinked and induced dECM-incorporated cell spheroids inside the cavities. The printed structures were cultured in William medium (Thermo Fisher Scientific) supplemented with hepatocyte maintenance supplements (CM 4000; Thermo Fisher Scientific) and 10% v/v fetal bovine serum (Capricorn, Germany) at 37 °C under 5% CO_2_.

Images of the printed spheroids were captured using a microscope (Leica, Germany), and ImageJ software (NIH, United States) was used to measured area, perimeter, and length of each spheroid. The circularity of the printed spheroids was calculated using the followed equation:

$$Circularity=4\pi \times \frac{\left[Area\right]}{{\left[Perimeter\right]}^{2}}$$

The aspect ratio was calculated as the ratio of the longest length to the shortest length of the printed spheroid.

### 2.5 Cell viability assay

Cell viability of the dECM-incorporated PMH spheroids was determined using a LIVE/DEAD viability kit (Thermo Fisher Scientific). The printed spheroids were washed using PBS and then incubated with 0.5 μg/mL of calcein AM and 2 μg/mL of ethidium homodimer-1 solution in PBS. Fluorescence images were acquired using a laser scanning confocal microscope (LSM780N; Zeiss, Germany).

### 2.6 Analysis of hepatic cell functionality

To assess the hepatic functionality of the dECM-incorporated PMH spheroids, albumin and urea secretion, Cytochrome P450 1A2 enzyme activity and gene expression were measured. Secreted albumin and urea from 20 PHM spheroids were quantified using a mouse albumin ELISA kit (Koma biotec, Republic of Korea) and a QuantiChrom urea assay kit (BioAssay Systems, United States), respectively. During the culture period, the culture media were collected every 24 h and analyzed according to the manufacturer’s instructions.

After 7 days of culture, P450-Glo™ CYP1A2 Assay System (Promega, United States) was used to analyze CYP1A2 activation of 10 of PHM spheroids. Briefly, the printed structures were incubated with 2 μM of 3-methylchoranthrene (3MC; Sigma) in the culture medium for 48 h for induction. Control groups were cultured without 3 MC. Then, 0.5 μM Luciferin1A2 solution with 3 mM salicylamide (Sigma) in PBS was added to each sample and incubated at 37°C for 30 min 25 μL of the incubated medium was collected into 96-well plates. Subsequently, 25 μL of luciferin detection reagent was added to the wells and incubated for 20 min at room temperature. After incubation, luminescence was measured using a multi-mode plate reader (Bio-Tek, United States) and normalized with respect to the value of the control group.

For quantitative real-time polymerase chain reaction (qRT-PCR), RNA was extracted from 50 PMH spheroids using easy-BLUE™ Total RNA Extraction Kit (iNTRON Biotechnology, Republic of Korea) and quantified using NanoDrop (Thermo Fisher Scientific). Helixcript™ Thermo Reversed Transcriptase (Nanohelix, Republic of Korea) was used to synthetase cDNA from the isolated RNA using manufacturer’s instructions. Gene expression was analyzed with Roche SYBR Lightcycler 480 SYBR Green I Master (Roche Diagnostics, Germany) using a light cycler 480 II (Roche Diagnostics). The primer sequences for hepatic function analysis are described in Supplementary Table S1. Gene expression levels were normalized relative to GAPDH using the ∆∆Ct method.

### 2.7 Immunofluorescence staining

Immunostaining was conducted to analyze the distribution of ECM components in the spheroid and hepatic function. The printed structures were fixed using 4% v/v paraformaldehyde in saline for overnight at 4°C. Tissue processing with serial concentration of EtOH was followed by paraffine embedding. The samples were sectioned into 7 μm thick sections using a microtome. After deparaffinization and antigen retrieval, the slides were immersed in a 5% w/v BSA solution for 1 h at room temperature. After blocking, anti-collagen I antibody (Abcam, UK), anti-laminin antibody (Novus, United States) and anti-glutamine synthetase (GS) antibody (Abcam) diluted in the BSA solution were applied for overnight at 4 °C. Following the primary antibody incubation, slides were washed using PBST, and then goat anti-mouse IgG H&L Alexa Fluor 488 (Thermo Fisher Scientific) and goat anti-rabbit IgG H&L Alexa Fluor 568 (Thermo Fisher Scientific) diluted in PBS were applied for 1 h at room temperature. After wash with PBST, nucleic acids were stained using 4′,6-diamidino-2-phenylindole (DAPI) for 5 min. Finally, the samples were washed with PBST, and fluorescence images were analyzed using a laser scanning confocal microscope (LSM780N; Zeiss).

### 2.8 Hepatotoxicity assay

A toxicity test was conducted to analyze sensitivity to hepatotoxic drugs of PMH spheroid group (without dECM) and PMH + dECM spheroid group (with 0.5% w/v of dECM) after 7 days culture. For each condition, ten spheroids were used in toxicity test. Stock solutions of acetaminophen (APAP; Sigma), amiodarone (Sigma), and celecoxib (Sigma) were prepared using DMSO (Sigma) and diluted into culture medium. A non-treated group was prepared, which contained the same concentration of the DMSO vehicle without any drugs. After treating each drug for 24 h, the viability of the structure was calculated using the CellTiter-Glo 3D Cell Viability Assay Kit (Promega, United States) according to manufacturer’s instructions. Briefly, structures were incubated in 100 μL of culture medium with 100 μL of CellTiter Glo reagent at room temperature for 30 min with gentle shacking. After incubation, luminescence was measured using a multi-mode plate reader (Bio-Tek) and normalized to the value of the non-treated group. The dose-response curves were plotted, and IC50 values were calculated using GraphPad Prism.

### 2.9 Statistical analysis

The data are presented with average values and standard deviation (SD). Data analysis was performed with GraphPad Prism. For statistical analysis, comparison between groups was conducted by one-way analysis of variance (ANOVA) followed by a Tukey’s multiple comparison test. Statistically significant differences between two groups were analyzed using Student’s t-test. Representative images acquired by the microscope were used for image analysis.

## 3 Results

### 3.1 Biochemical characterization of the dECM for cell bio-ink

The dECM-based cell bio-ink and matrix ink were prepared, and a 3D bioprinter with multi-head dispensing modules was used to produce the dECM incorporated cell spheroid (Figure 1). To prepare the dECM-based cell bio-ink, porcine liver was decellularized and residual biochemicals were analyzed (Supplementary Figure S1). After decellularization, the liver tissue color became white, and H&E staining results confirmed that most of the cellular components in the issue were removed, while the ECM components were well preserved (Supplementary Figure S1A). Quantitative assay results showed that 96.13% DNA in the tissue was removed through decellularization process, and 55.67% GAGs and 441.84% collagen were remained (Supplementary Figure S1B-D). As shown in the data, the preserved collagen concentration was increased after decellularization. This is because the cellular components within the liver tissue have been removed, resulting in a relative increase in the collagen proportion of the ECM. Similar trends were observed in other studies (Wu et al., 2015; Xu et al., 2023). After decellularization, the liver dECM solution was mixed with the sacrificial bio-ink to prepare dECM-based cell bio-ink.

### 3.2 Mechanical property and printability analysis of the cell bio-ink

**FIGURE 2:**
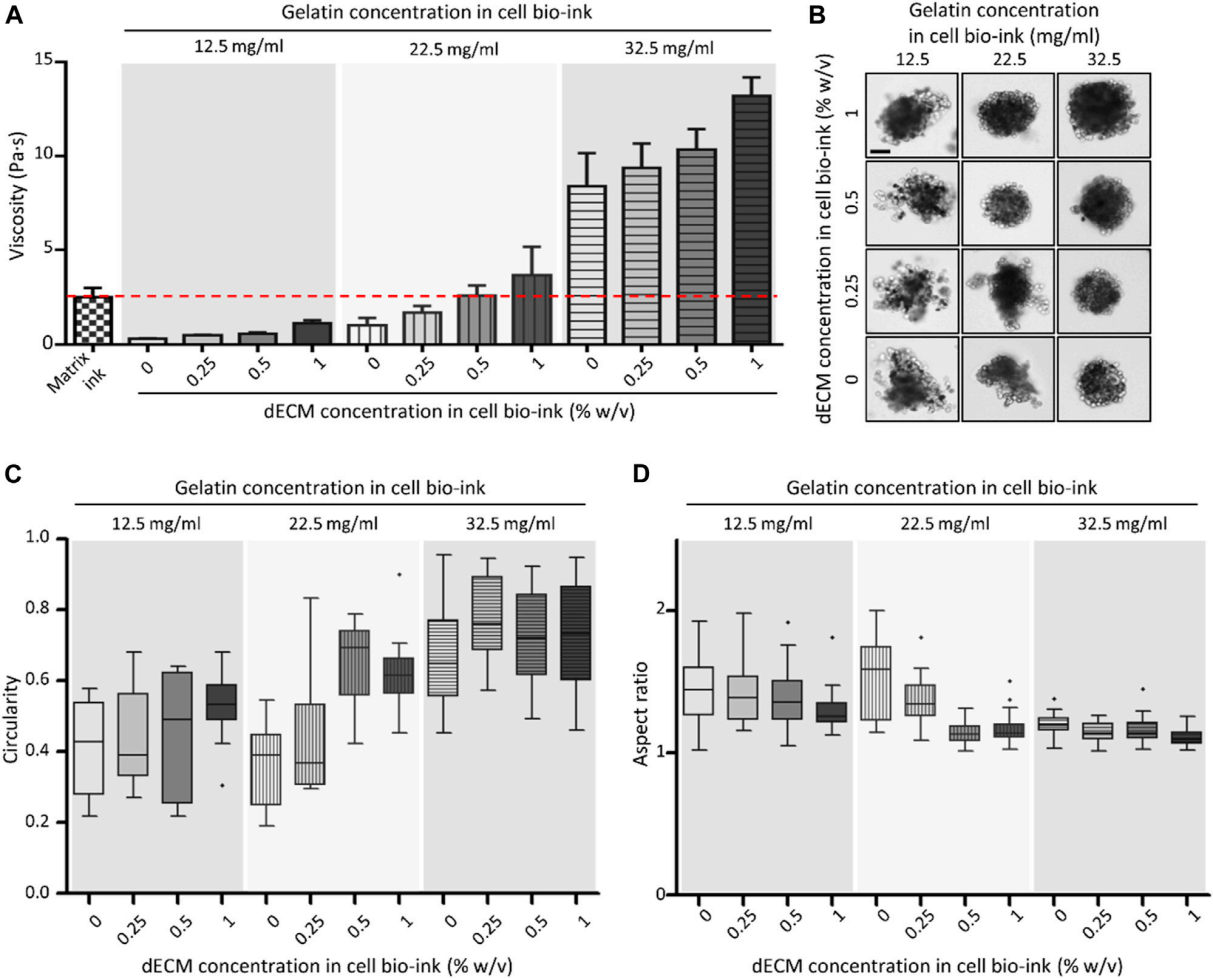
**V**iscosity and spheroid printing results of dECM-based cell bio-ink. **(A)** Viscosity of matrix ink and dECM-based cell bio-ink in the variation of gelatin and dECM concentrations (n = 3). The red line indicated the viscosity of the matrix ink. **(B)** Microscopy images of primary mouse hepatocyte (PMH) spheroids bioprinted with dECM-based cell bio-ink (Scale bar = 100 μm). Measured circularity **(C)** and aspect ratio **(D)** of the dECM-incorporated PMH spheroids (n = 20).

Rheological properties and printability were evaluated according to changes in composition of the dECM-based cell bio-ink. The (Supplementary Figure S2) demonstrated shear sweep analysis results of the bio-ink. The viscosity of all dECM-based cell bio-ink groups and matrix ink decreased with increasing shear rate. This shear thinning behavior was essential characteristic for bio-ink to reduce the cell damage during the printing process. The viscosity of the bio-inks at a shear rate of 20 1/s were estimated in Figure 2A. The viscosity of dECM-based cell bio-inks increased by higher gelatin and dECM concentration. As shown in the figure, in the groups with 22.5 mg/mL of gelatin, the viscosity of the dECM-based cell bio-ink (with dECM ≥ 0.5% w/v) was similar to that of the matrix ink. At a gelatin concentration of 32.5 mg/mL, all of the cell bio-ink groups had a higher viscosity than that of the matrix ink.

The concentration of gelatin and dECM in cell bio-ink also directly affected the shape of the printed dECM-incorporated hepatocyte spheroids. As shown in the Figure 2B, printed cell spheroids tended to have a uniform and round shape when the viscosity of the cell bio-ink was similar to or higher than that of the matrix ink. Especially, at a gelatin concentration of 32.5 mg/mL, spheroids exhibited a good round shape at all dECM concentration groups. The circularity and aspect ratio of printed spheroids were calculated based on the image data (Figures 2C,D). As shown in the Figure 2C, overall, as the gelatin and dECM concentration within the cell bio-ink increased, the circularity of the printed cell spheroid shape increased. In the groups with a gelatin concentration of 32.5 mg/mL, dECM incorporated cell spheroids with an average circularity of more than 0.67 were produced in all dECM concentration conditions. Similarly, the aspect ratio of the printed cell spheroids approached value one as the gelatin and dECM concentration increased (Figure 2D). In the cell bio-ink group containing 32.5 mg/mL gelatin, the average aspect ratio was close to one, and the standard deviation values were relatively small compared to the other groups. These results confirmed that cell bio-ink with viscosity similar or higher than matrix ink was available to print circular-shaped cell spheroids. Based on the test results, a cell bio-ink containing 32.5 mg/mL gelatin was used in further experiments.

### 3.3 Characterization of dECM-incorporated spheroids

**FIGURE 3:**
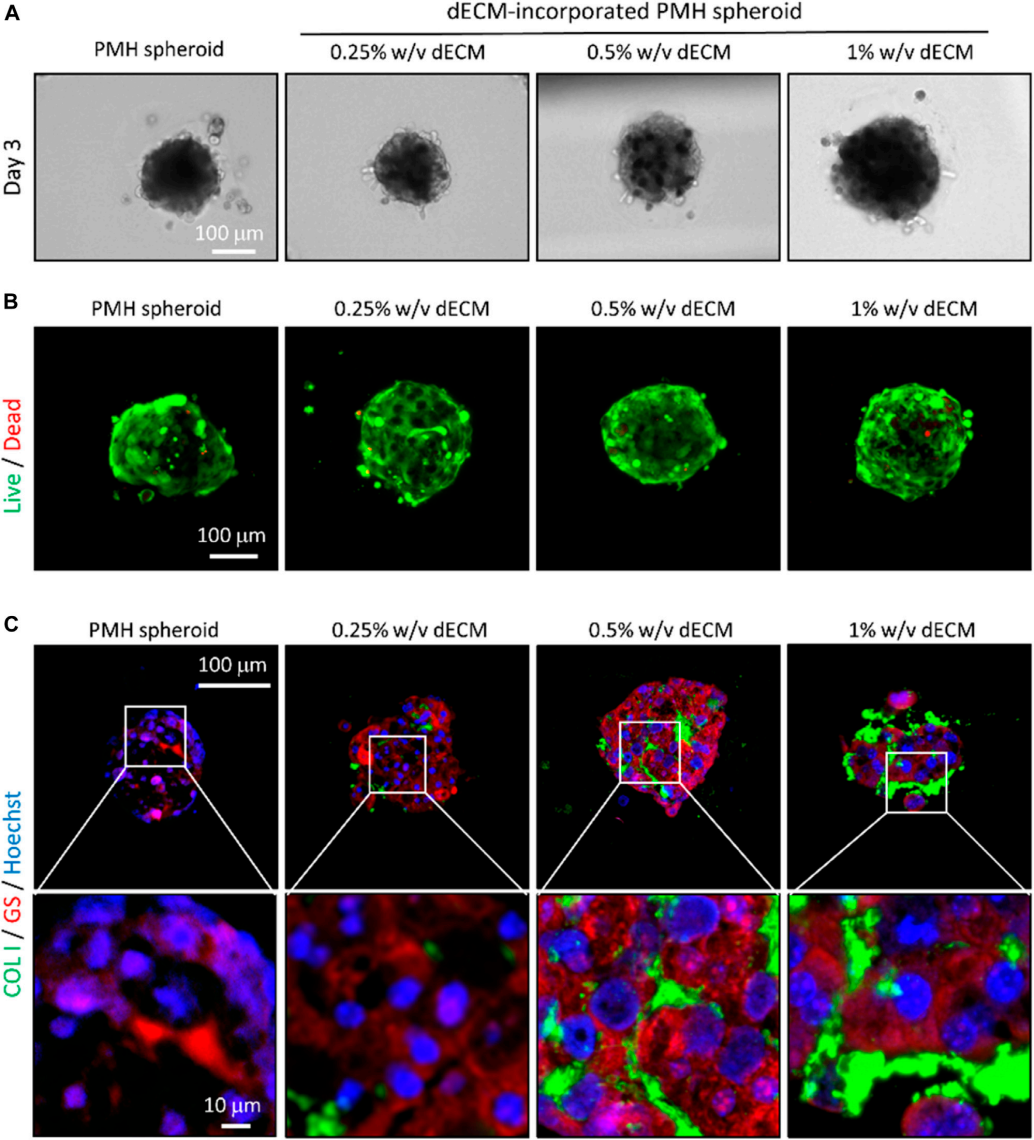
Bioprinted dECM-incorporated PMH spheroids in the variation of dECM concentration. **(A)** Microscopy images of bioprinted, dECM-incorporated PMH spheroids in the variation of dECM concentration. Live/dead- **(B)** and immuno-staining **(C)** results of the bioprinted spheroids on day 3 after printing. (COL1: Collagen type 1, GS: Glutamine synthetase).

The dECM concentration of cell bio-ink directly affected the size of spheroids and distribution of the dECM inside spheroids. After 3 days of printing, uniform and compact dECM-incorporated cell spheroids were formed in all dECM concentration groups (Figure 3A). Although the same amount of the cell bio-ink was printed, the diameter of the printed PMH spheroids slightly increased as the dECM concentration increased (Supplementary Figure S3). In the 0.25% w/v dECM group, cell spheroids with a diameter of 160 µm were fabricated. Besides, the diameter of spheroids increased to approximately 220 μm at the dECM concentration of 1% w/v. Compared with PMH only spheroids, the spheroids in the 0.25% w/v dECM had a decrease in diameter of about 8.84%. This result can be interpreted as dECM influencing the formation of more compact spheroids during the spheroid induction. (Supplementary Figure S4 **)** showed morphological change of the printed spheroids during 14 days. The spheroids were compacted after printing and maintain their circular shape during culture period. The viability of cell spheroids according to dECM concentration was evaluated through live/dead staining (Figure 3B). Staining results showed that the majority of cells were stained green, indicating a very high level of viability, and red stained dead cells were rarely observed even in the center of the spheroids after 3 days culture. Likewise, the Cell TiterGlo viability assay results showed similar values between the groups, indicating that there is no significant difference **(** Supplementary Figure S4B, C **)**. Followed by two-step crosslinking with CaCl_2_ and temperature, the crosslinked dECM hydrogel remained within the printed spheroids. The distribution of dECM within the spheroids was analyzed through immunostaining results of collagen, a representative component of dECM (Figure 3C). The staining results showed that dECM was thinly distributed between the cells within the spheroids bioprinted with dECM-based cell bio-ink. According to dECM concentration, the dECMs were distributed as thicker layer between the cells. In addition, GS which was one of the representative hepatocyte markers for ammonium homeostasis was confirmed through immunostaining. Especially, 0.25% w/v and 0.5% w/v dECM groups had higher expression than 0% w/v and 1% w/v dECM groups.

### 3.4 Enhanced hepatic functionality of dECM-incorporated spheroids

**FIGURE 4:**
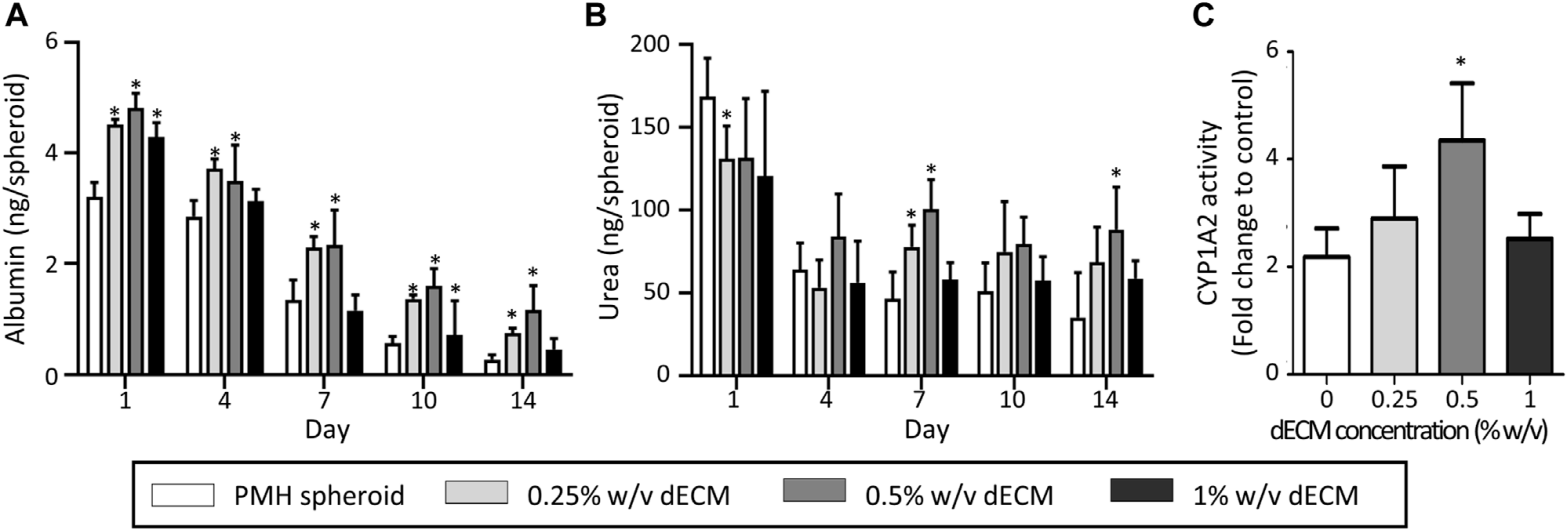
Hepatic functions of dECM-incorporated PMH spheroid in the variation of dECM concentration. Albumin **(A)** and urea **(B)** secretion of dECM-incorporated PMH spheroids in the variation of dECM concentration for 14 days after printing. **(C)** CYP1A2 activity in response to 3-MC on day 7. (n = 5, *p < 0.05 *versus* PMH spheroid group).

The dECM concentration also affected the hepatic functionalities of PMH spheroids, including albumin secretion, urea secretion, and Cyp1a2 activity (Figure 4). As a result of albumin analysis, all dECM incorporated PMH spheroid groups showed higher albumin secretion levels than PMH only spheroid group (Figure 4A). As the dECM concentration increased, the albumin secretion level increased but 1% w/v dECM group. The 0.5% w/v dECM group demonstrated the highest albumin secretion level, with a 4.28-fold increase compared to the PMH spheroid group on day 14. A similar trend was observed in the urea secretion analysis (Figure 4B). As the dECM concentration increased, the amount of urea secretion also increased up to 0.5% w/v. The 0.5% w/v dECM group showed the highest urea concentration and 2.51-fold secretion compared to the PMH spheroid group on day 14. Finally, Cyp1a2 activity of the dECM incorporated PMH spheroids was measured on day 7 (Figure 4C). The Cyp1a2 activity also enhanced by dECM concentrations up to 0.5% w/v. The 0.5% w/v dECM group performed a 1.99-fold higher level than that of the PMH spheroid group. Based on these results, the 0.5% w/v dECM-incorporated PMH spheroid (PMH + dECM spheroid) group was selected for further experiments as the highest enhancement of PMH functionality.

**FIGURE 5:**
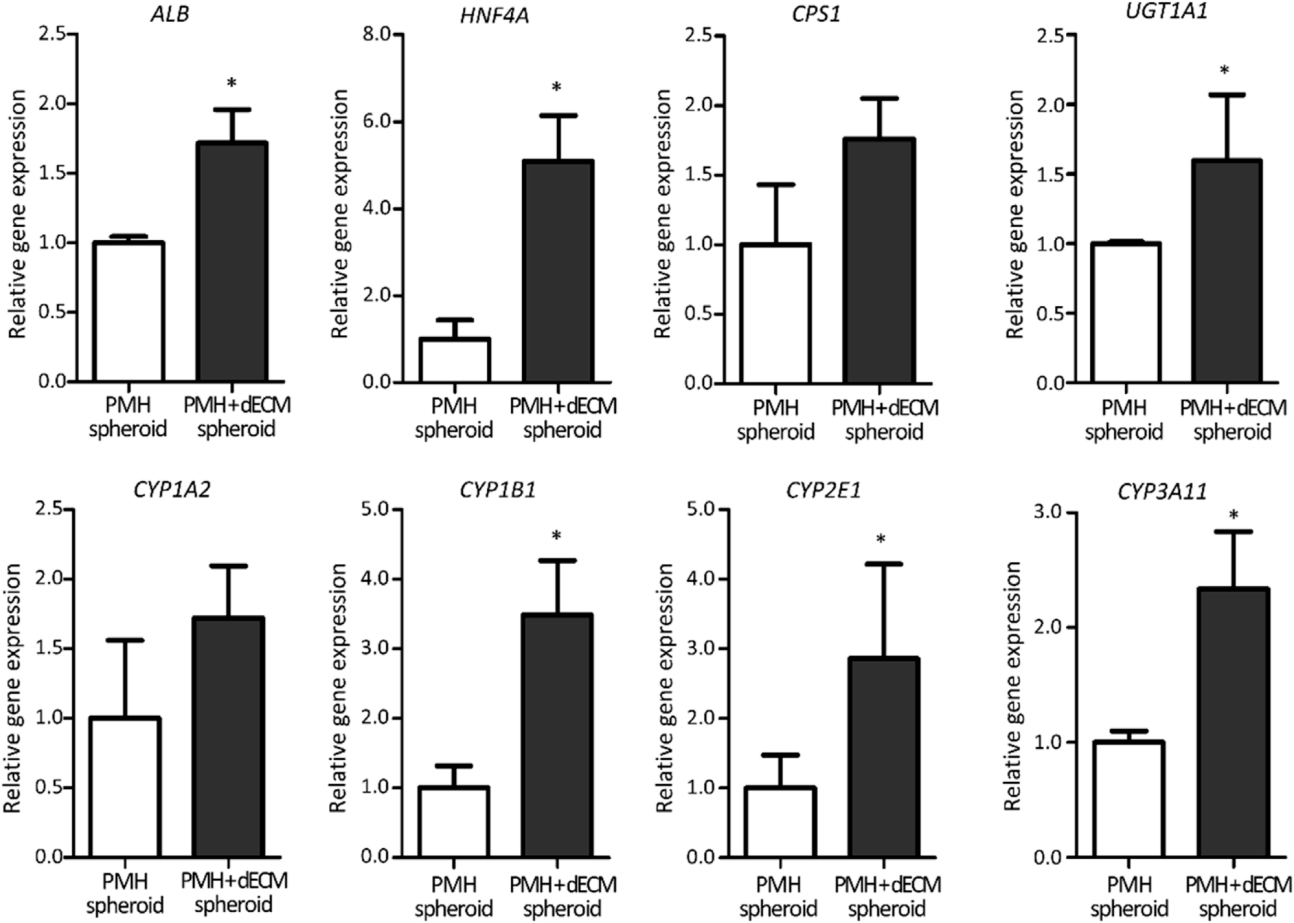
Hepatic gene expression level of dECM-incorporated PMH spheroids (PMH + dECM spheroid). Relative expression of liver detoxification and metabolism related genes in PMH and PMH + dECM spheroids. Assay was performed on day 7 and the transcriptional levels were normalized by using GAPDH (n = 4, *p < 0.05 *versus* PMH spheroid group).

The hepatic functionality of the PMH + dECM spheroid was confirmed again through qRT-PCR (Figure 5). The qRT-PCR analysis results of liver detoxification and metabolism markers indicated that the PMH + dECM spheroid group had enhanced gene expression levels compared to the PMH spheroid group. In measured all markers, the PMH + dECM spheroid group showed approximately 1.60- to 5.09-fold higher gene expression levels compared to the PMH spheroid group. Among them, statistical significance was also observed for *Alb*, *Hnf4a*, *Cps1*, *Ugt1a1*, *Cyp1b1*, *Cyp2e1*, and *Cyp3a11* markers.

Hepatic functions of PMH spheroids were greatly influenced by the composition of the incorporated ECM. To investigate this aspect, collagen-incorporated spheroids were prepared in this study. Supplementary Figure S5 shows the results of comparing the hepatic functionality of dECM- and collagen-incorporated spheroids (PMH + dECM and PMH + COL spheroid). Similar to the PMH + dECM spheroid, PMH + COL spheroid was also printed in a spherical shape and formed a compact spheroid on day 3 Supplementary Figure S5A. Live/dead staining images showed that most cells were alive Supplementary Figure S5B. Immunostaining for collagen and laminin, which were representative components of dECM, was performed (Supplementary Figure S5C). The staining results showed that the collagen and laminin was well observed in the PMH + dECM spheroid group, whereas laminin was not observed in the PMH + COL spheroid group. These biochemical composition differences influenced the albumin and urea secretion of hepatocytes Supplementary Figure S5D, E. During culture period of 14 days, both albumin and urea secretion were improved in the PMH + dECM spheroid group than PMH + COL spheroid group. On day 14, albumin secretion increased by 2.42-fold, and urea secretion increased by 1.67-fold compared to the PMH + COL spheroid group.

### 3.5 Hepatotoxic drug test using dECM-incorporated spheroids

**FIGURE 6:**
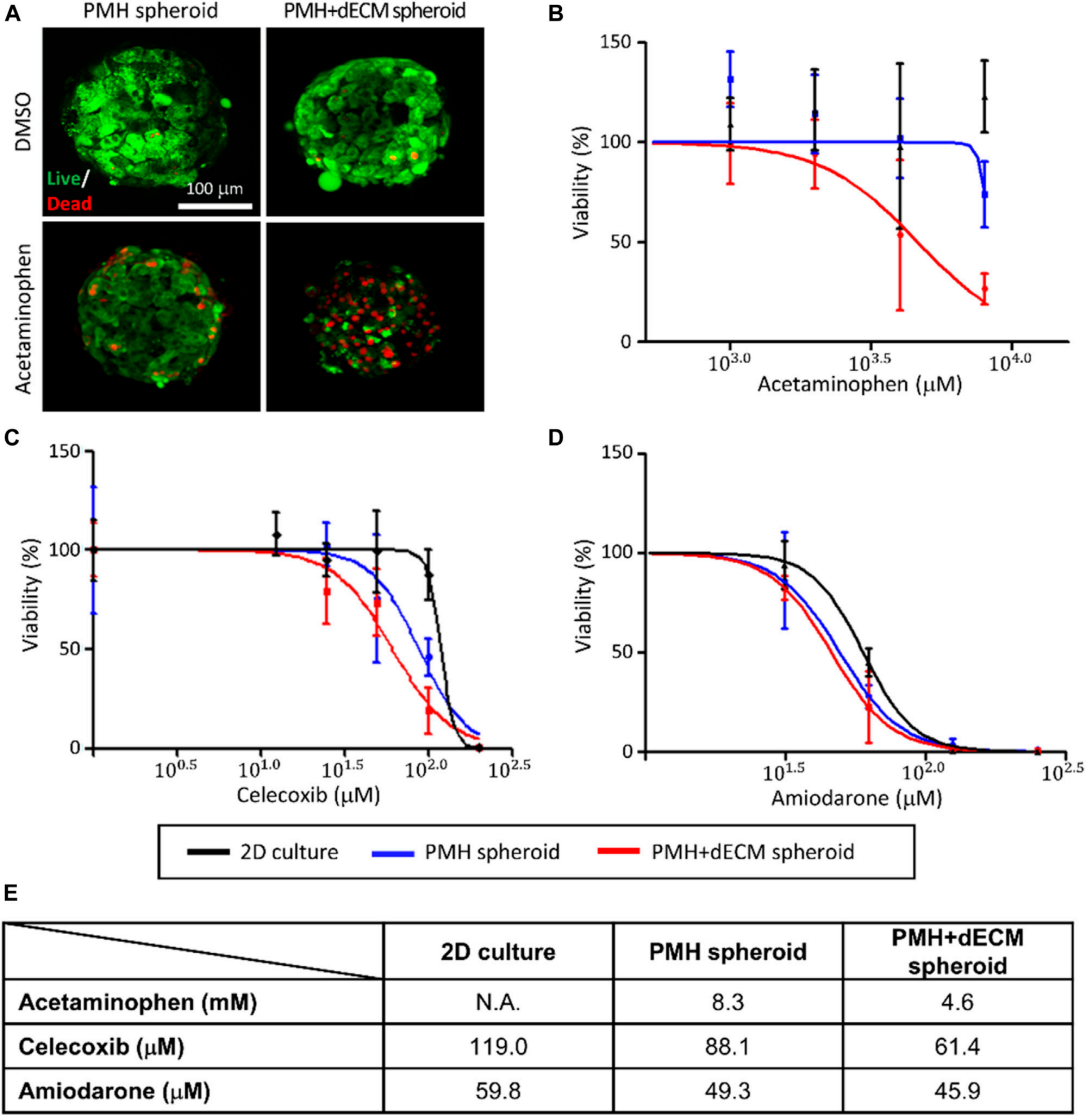
Hepatotoxic drug test results of dECM-incorporated PMH spheroids. **(A)** Live/dead staining results of PMH spheroid group (without dECM) and PMH + dECM spheroid group (with 0.5% w/v dECM). After 7 days culture, the spheroids were treated with 8 mM acetaminophen for a day and stained using calcein AM (live) and ethidium homodimer-1 (dead). Measured viability of the PMH and PMH + dECM spheroids after treatment of various concentration of hepatotoxic drugs; acetaminophen **(B)**, celecoxib **(C)** and amiodarone **(D)**. **(E)** Calculated IC50 of the spheroids for the hepatotoxic drugs. The IC50 was calculated using the drug concentration-viability curves (**(B)**, **(C)**, and **(D)**).

Evaluation of the response of dECM-incorporated PMH spheroids to various hepatotoxic drugs was performed. After treatment with 4 mM APAP, firstly, live/dead staining was performed (Figure 6A). The staining results showed that when treated with 4 mM APAP, the number of dead cells staining red increased in both groups. Among the two groups, a significantly higher percentage of dead cells was observed in the PMH + dECM spheroid group. Next, cell spheroids were treated with hepatotoxic drugs (APAP, celecoxib, and amiodarone) at different concentrations, and their viability was measured (Figures 6B–D). Viability of the spheroids decreased as the concentration of each drug increased in every groups. As shown in the graphs, among the three groups, the PMH + dECM group showed the highest sensitivity to drug-induced hepatotoxicity, and the 2D culture group had the lowest responsiveness. Based on the measured dose-dependent viability data, the IC50 values for each drug were calculated (Figure 6E). The results showed that the PMH + dECM spheroid group had the lowest IC50 values for the three drugs. The IC50 values of the PMH + dECM spheroid group were significantly lower than those of the 2D culture group, and when compared to PMH spheroid group they had IC50 values up to approximately 55% lower. These results demonstrate that the PMH + dECM spheroid had high sensitivity to hepatotoxic drugs and the spheroids can be used as a valuable *in vitro* model for drug testing.

## 4 Discussion

Traditional spheroid culture enhanced the viability and hepatic functionality of primary hepatocytes by reinforcing cell-cell interactions with non-adherent molding and hanging drop methods (Bell et al., 2016; Kyffin et al., 2019). Various studies have utilized hepatocytes spheroids to create liver tissue constructs and conduct toxicity tests using them (Yan et al., 2015; Mizoi et al., 2020). However, the conventional hepatocytes spheroid model has limitations in cell-ECM interaction, which is known to be one of the important factors in recreating the native tissue microenvironment. In this study, the dECM-incorporated cell spheroid printing method was developed and used to fabricate hepatocytes spheroid with improved cell-ECM interactions. These spheroids exhibited enhanced hepatic functionality by mimicking the biochemical environment of *in vivo* liver tissue.

The biomaterials in the bio-inks had good cytocompatibility and printability for 3D bioprinting. HA has proper cytocompatibility and used to increase uniformity of dispensing rate of the printed bio-inks (Kang et al., 2016). Alginate is also biocompatible material but lacked arginyl-glycyl-aspartic acid that was cell binding motif to mammalian cells (Augst et al., 2006). This characteristic induced self-assembly of primary hepatocyte spheroids when cell containing bio-inks were printed into alginate-based matrix ink. In addition, the concentrations of gelatin and dECM in the dECM-based cell bio-ink used for spheroid printing influenced the physical properties of the bio-ink and the shape of the printed spheroids. The rheological behavior by the gelatin and dECM concentrations in the cell bio-ink showed that viscosity increased as the concentrations increased. Particularly, an increase in gelatin concentration led to a significant enhancement of the bio-ink’s mechanical properties. Gelatin has the property of being thermally crosslinked at low temperatures. This property has been utilized to enhance the properties of bio-inks, and make it an important component for improving the printability of various bio-inks (Shin and Kang, 2018). Furthermore, an increase in the concentrations of dECM and gelatin in the cell bio-ink enabled the printing of spheroids with a more rounded and uniform shape. This trend was more obvious in the results of analyzing circularity and aspect ratio using bright-field images. Circularity indicated the roundness of spheroid surfaces, and aspect ratio describes the elongated state of spheroids. A perfectly round spheroid had values of one for both circularity and aspect ratio. On the other hand, as spheroid shapes became irregular and spread form, their values were higher than 1. The measurement results showed that the 22.5 mg/mL of gelatin groups with dECM concentrations more than 0.5% w/v and the 32.5 mg/mL of gelatin groups exhibited values close to 1. Especially, 32.5 mg/mL of gelatin groups were able to print homogeneous spheroids regardless of the dECM concentration, and making it the optimal concentration for spheroid printing.

The dECM-incorporated cell spheroids with various dECM concentrations displayed an increase in diameter as the concentration of dECM in cell bio-ink increased. This was interpreted as an effect of the increase in the volume of dECM contained within the spheroid. In contrast, the diameter of the 0.25% w/v dECM spheroid group was smaller than that of the PMH spheroid group without dECM. This phenomenon of forming more compact and circular spheroids when additives such as ECM proteins are present during spheroid formation has been observed similarly in other studies (Ivascu and Kubbies, 2006; Leung et al., 2015). It is known to result from the enhancement of cell-matrix interactions mediated by cell surface proteins during the spheroid formation process. Similarly, in this study, the proteins contained within the dECM hydrogel within the printed cell spheroid influenced the interaction with the surface proteins of PMHs, contributing to the formation of compact spheroids.

The concentration of dECM included in the cell bio-ink also had an impact on the hepatic functionality of the printed spheroid. Primary hepatocyte had experienced hepatic functionalities lost after isolation by dedifferentiation (Kiamehr et al., 2019; Sun et al., 2019). However, within the range of 0%–0.5% w/v dECM concentration, an increase in dECM concentration led to enhanced functional preservation of primary hepatocytes especially in albumin and urea secretion and CYP1A2 activity. However, the 1% w/v dECM spheroid group showed a tendency of decreased hepatic function. The immunostaining images of GS, which has well known role in homeostasis of ammonium (Freig et al., 2021), showed similar results. The decrease in hepatic functionality in this 1% w/v dECM group can be interpreted as being related to the distribution of dECM within the spheroid. The developed dECM-incorporated cell spheroids had distributed dECM hydrogel within the spheroid, and enhancing both cell-cell and -ECM interactions. However, when the concentration of dECM was too high, the dECM hydrogel tended to accumulate excessively between cells and inhibit the cell-cell interactions, which is one of the advantage of cell spheroid culture. Immunostaining results for collagen type I showed an increased stained range inside spheroids as dECM concentration increased. Especially, in the 1% dECM group, collagen was found to be positioned thickly between cells inside spheroids, and creating separation between them. Therefore, the thick dECM hydrogel within the 1% w/v dECM spheroid hindered cell-cell interactions, resulting in decreased hepatic functionality. Similarly, Tao et al. observed that a high concentration of Matrigel within spheroids had decreased hepatic functionality because ECM hydrogel hindered cell-cell interactions (Tao et al., 2020). In conclusion, it was confirmed that the 0.5% w/v dECM-incorporated spheroid group was the optimal condition for hepatocytes spheroid printing. Although the hepatic functionality analysis was conducted or 14 days in this study, the long-term study for such as chronic drug induced toxicity test will be needed in further study.

In this study, hepatocytes spheroids were printed using a dECM based cell bio-ink to enhance functionality. Biomaterials used in the fabrication of bioartificial liver are important factors for improving functionality by providing a suitable biochemical environment for cells (Kaur et al., 2020). Therefore, various materials, such as collagen, Matrigel, and fibrinogen, are commonly used for the fabrication of bioartificial livers (Hoshiba et al., 2006). However, native tissues are consist of various components that interact to maintain metabolism. Therefore, it is known that liver functionality is enhanced when using native ECM-like materials rather than homogeneous materials (Lang et al., 2011). dECM can mimic tissue-specific biochemical environments because it retains various native ECM materials. According to research by Han et al., proteomic analysis of dECM derived from the liver, heart, cornea, and skin revealed specific proteins unique to each tissue, and especially, liver dECM contains several components which were not found in other tissues (Han et al., 2019). Similarly, the liver dECM used in this study had ECM components such as collagen and GAGs, while most of its cell components were removed after decellularization. Spheroids printed using the prepared liver dECM showed homogeneous distribution of representative dECM components, collagen, and laminin, was confirmed by immunostaining. Collagen and laminin are well-known components of the liver ECM, and these components are essential for the survival and differentiation of hepatocytes and liver tissue organization (Oda et al., 1995; Cameron et al., 2015; Grant et al., 2019). Although we analyzed only collagen and laminin, it is expected that other dECM components were also preserved in the spheroids and effectively mimicking the physiological environment of liver tissue. These heterogeneous biochemical components in dECM-incorporated cell spheroids also influenced the hepatic functionality of the spheroids. A comparison of hepatic functionality based on the biomaterials used for spheroid printing revealed that functionality was enhanced when dECM was used compared to when homogeneous collagen was used. This suggests that the various liver-specific biochemicals contained in dECM enhanced cell-ECM interactions, and contributing to the maintenance of PMHs functionality. Therefore, when utilizing this printing process, tissue-specific bio-inks could be expected to be used for engineering not only liver tissue but also various functional tissue units.

Finally, the feasibility of dECM-incorporated cell spheroids as a drug testing platform was confirmed through responsiveness analysis to hepatotoxic drugs. In drug development process, several clinically approved drugs had reported chronic hepatic failure such as fialuridine for hepatitis B virus (McKenzie et al., 1995). Therefore, highly sensitive hepatocyte model that can predict hepatotoxicity is necessary to be developed (Bell et al., 2016). Various researches developed drug testing models with hepatotoxins sensitivity, particularly hepatocytes spheroid cultures have demonstrated a higher sensitivity to toxic compounds (Proctor et al., 2017). Vorrink et al. utilized primary human hepatocytes spheroids for toxicity testing involving 123 drugs, and the results showed that they detecting 69% of hepatotoxic compounds without any false positive results (Vorrink et al., 2018). However, traditional hepatocytes spheroids used for toxicity testing have limitations in reproducing the biochemical microenvironment of liver tissue. The developed dECM-incorporated cell spheroids exhibited increased sensitivity to hepatotoxic drugs based on their improved functionality compared to traditional spheroids without dECM incorporation. The viability of the dECM-incorporated cell spheroid group decreased more rapidly in response to hepatotoxic drugs, including acetaminophen, celecoxib, and amiodarone, compared to the PMH spheroid and 2D groups. The drugs used in the experiments caused liver toxicity due to the direct effects of the drugs and their biotransformation metabolism (Van Summeren et al., 2013; Syed et al., 2016). Therefore, when hepatocyte drug metabolism becomes more active, sensitivity to hepatotoxic drugs was increased (Takayama et al., 2014). The improved drug metabolism in dECM-incorporated spheroid group increased drug sensitivity. CYP1A2, one of the representative drug metabolism enzymes was highly expressed in the 0.5% w/v dECM group compared to PMH spheroid group. Consequently, the developed dECM-incorporated cell spheroids can be utilized as an *in vitro* liver model for drug-induced liver toxicity test platform with enhanced sensitivity compared to traditional methods.

In this way, the dECM-based hepatocyte spheroid bioprinting technology developed in this study has demonstrated the potential to create high-performance hepatic models and apply them to drug screening platforms. This technology, utilizing precision printing techniques, allows for the production of reproducible drug screening platforms through automated processes. Furthermore, although the current research has focused on liver models, it possesses a high degree of process flexibility that can be applied to various types of tissue models. Consequently, it will be highly useful for the development of multi-organ models alongside various tissue models. Moreover, this technology holds significant potential for the development of liver tissue regeneration techniques for the treatment of liver failure.

## 5 Conclusion

In this study, we fabricated novel dECM-incorporated hepatocyte spheroid to enhance both cell-cell and -ECM interactions simultaneously. The complex biochemical composition of dECM properly mimicked *in vivo* liver tissue’s biochemical environment inside the printed spheroids and leading to increased cell-ECM interaction. Additionally, we demonstrated that the dECM-incorporated PMH spheroids exhibited enhanced hepatic functionality and increased sensitivity to hepatotoxic drugs compared to the PMH spheroid group without dECM. These results showed that the potential of dECM-incorporated spheroid printing technology as an advanced method for highly functional *in vitro* liver tissue engineering.

## Data Availability

The original contributions presented in the study are included in the article/Supplementary Material, further inquiries can be directed to the corresponding author.
