# Supplementary material for: 3D bioprinting of dECM-incorporated hepatocyte spheroid for simultaneous promotion of cell-cell and -ECM interactions
Source: Front Bioeng Biotechnol. 2023 Nov 13;11:1305023. doi: 10.3389/fbioe.2023.1305023 (PMC10679743; doi:10.3389/fbioe.2023.1305023)
Supplement: Supplementary file 1 [file DataSheet1.docx]

*Supplementary Material*

**3D bioprinting of dECM-incorporated hepatocyte spheroid for simultaneous promotion of cell-cell and -ECM interactions**

Min Kyeong Kim^1,2,^*^†^*, Wonwoo Jeong^1,3,^*^†^*, Seunggyu Jeon^1,3^, and Hyun-Wook Kang^1,*^

^1^Department of Biomedical Engineering, Ulsan National Institute of Science and Technology, Ulju-gun 44919, Ulsan, Republic of Korea

^2^Center for Scientific Instrumentation, Korea Basic Science Institute, Cheongju 28119, Chungbuk, Republic of Korea

^3^Wake Forest Institute for Regenerative Medicine, Wake Forest University School of Medicine, Winston-Salem, NC 27157, USA

*^†^*These authors contributed equally to this work.

*** Correspondence:***Hyun-Wook Kang, Ph.D.*

*Department of Biomedical Engineering, Ulsan National Institute of Science and Technology*

*50, UNIST-gil, Ulsan 44919, South Korea*

*Tel.: +82 52 217 2527; E-mail address:* [*hkang@unist.ac.kr*](mailto:hkang@unist.ac.kr)

**Supplementary Table**

**Table S1. Primer sequences used for primary mouse hepatocyte**

| **Gene** | **Forward (5'-3')** | **Reverse (5'-3')** |
| --- | --- | --- |
| *ALB* | AGCCCACTGTCTTAGTGAGG | TCTTGCACACTTCCTGGTCC |
| *HNF4A* | CTAACACGATGCCCTCTCAC | GCAGGAGCTTGTAGGATTCAG |
| *CPS1* | AAGTAGAGATGGACGCTGTTG | CTTGGCTGATGGTCTGTGTAG |
| *UGT1A1* | AGATTACCCCAGGCCCATC | ATGGCTTTCTTCTCCGGAAT |
| *CYP1A2* | ATAACTTCGTGCTGTTTCTGC | ACCGCCATTGTCTTTGTAGT |
| *CYP1B1* | ATTCTCAGTGGGCAAACGG | GGATTCTAAACGACTTGGGCT |
| *CYP2E1* | GGAATGGGGAAACAGGGTAAT | GCACAGCCAATCAGAAAGGT |
| *CYP3A11* | TGGTCAAACGCCTCTCCTTGCTG | ACTGGGCCAAAATCCCGCCG |
| *GAPDH* | TGCCCCCATGTTTGTGAT | TGTGGTCATGAGCCCTTC |

**Supplementary Figures**

**
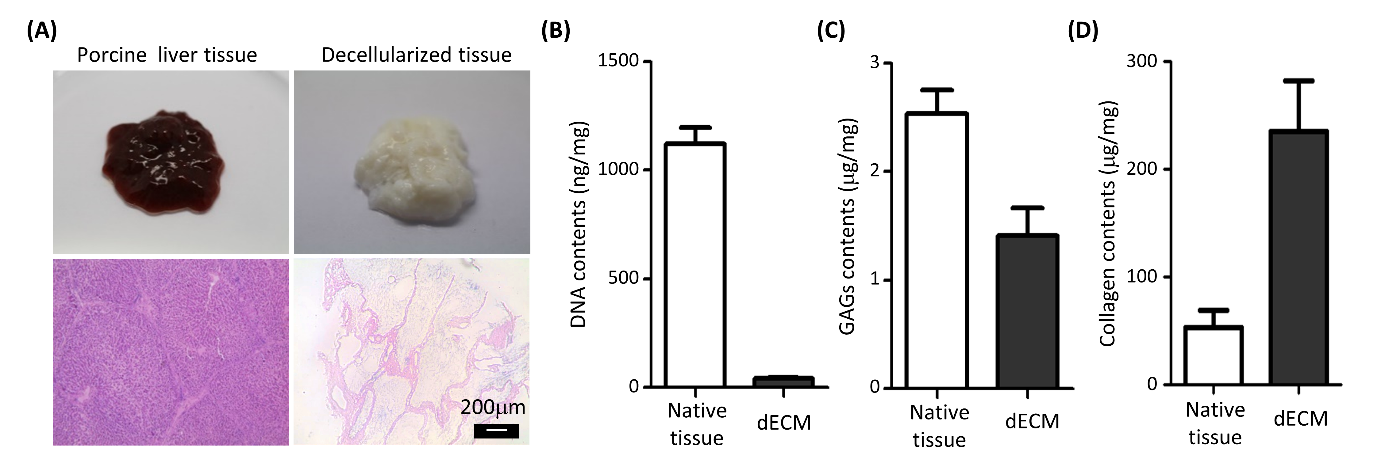
**

**Supplementary Figure 1. Characterization of dECM materials.** (A) Gross view (upper) and H&E staining results (lower) of native and decellularized porcine liver tissues. Measured DNA (B), GAGs (C) and collagen (D) contents of the native and dECM material.

**
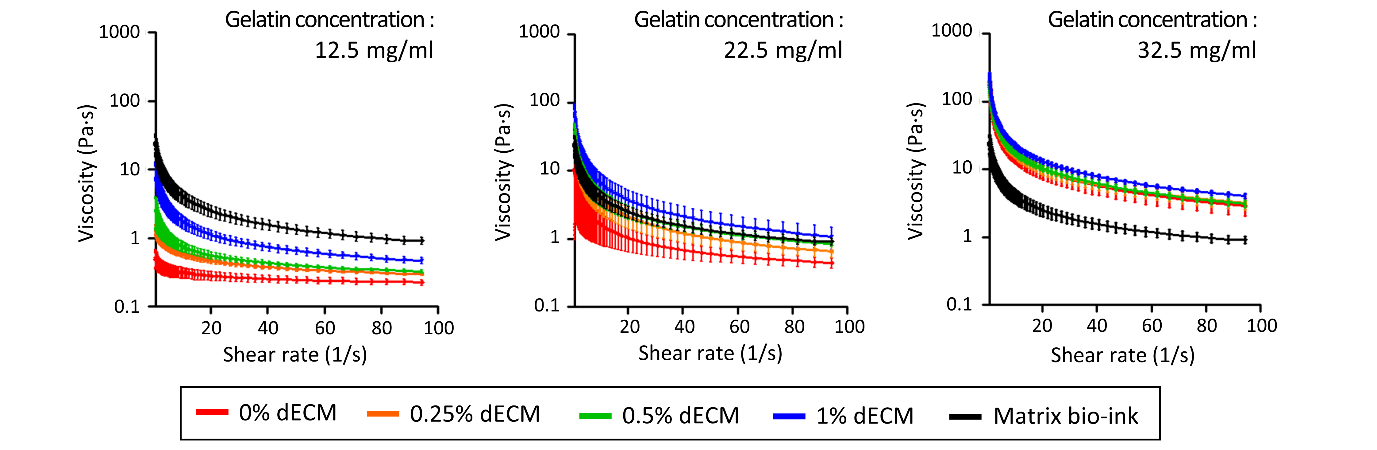
**

**Supplementary Figure 2. Rheological property of dECM-based cell bio-inks and matrix ink.** Viscosities of the dECM-based cell bio-inks (gelatin concentration: 12.5 mg/ml, 22.5 mg/ml, 32.5 mg/ml) and matrix ink were measured according to the change in shear rate (n=3).

**
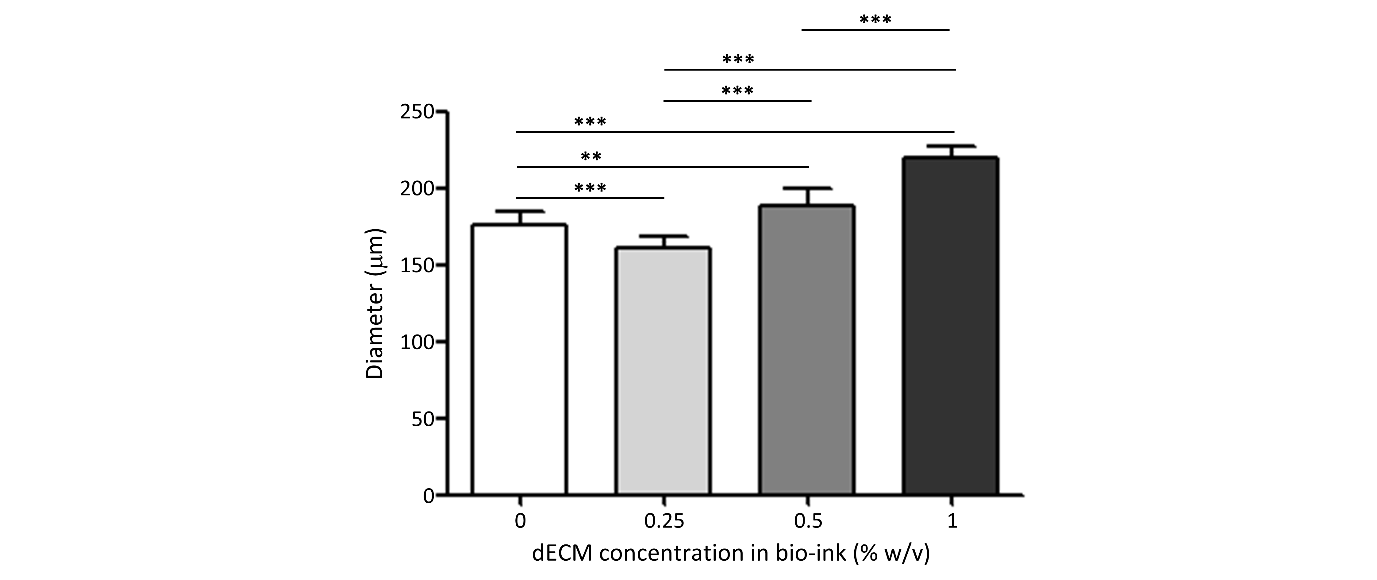
**

**Supplementary Figure 3. Measured diameters of bioprinted, dECM-incorporated PMH spheroids in the variation of dECM concentration.** After printing, the diameters were measured on day 3 (n=20, **p < 0.01, ***p < 0.001).


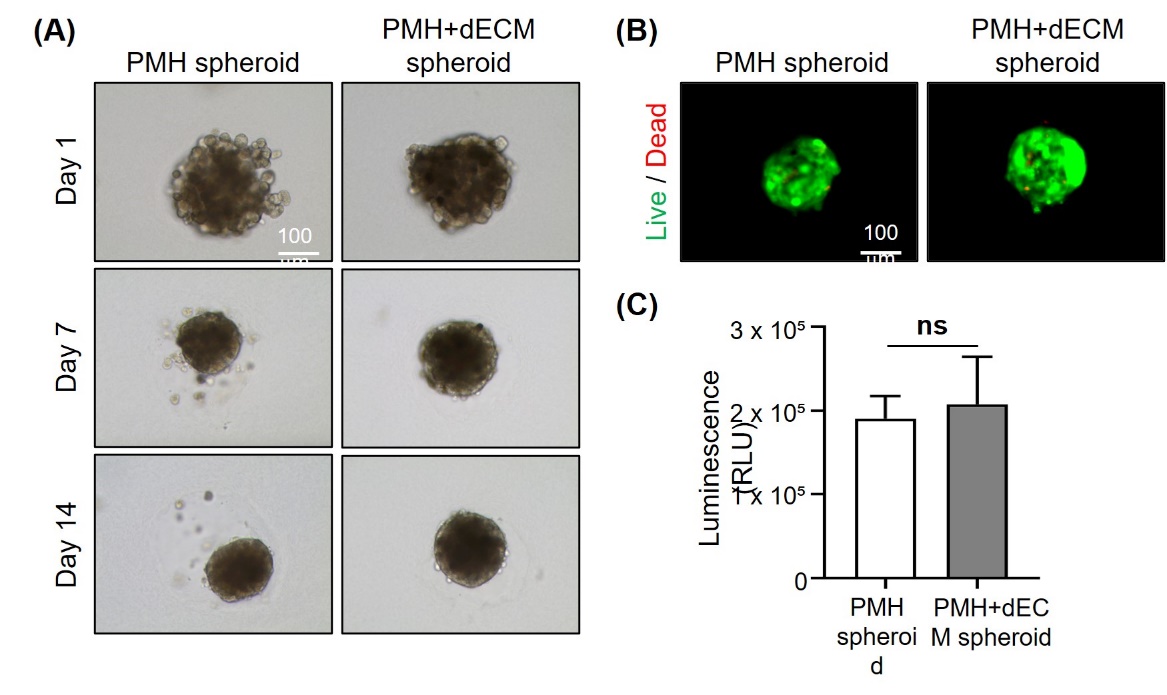


**Supplementary Figure 4. Morphology and viability of the bioprinted dECM-incorporated PMH spheroids.** Optical microscope images of PMH spheroid group and 0.5% w/v dECM group during 14 days culture (A). The live/dead staining (B) and CellTiter-Glo cell viability assay (C) of PMH spheroid group and 0.5% w/v dECM group was conducted on day 7 after printing (n=3, ns: not significant).

**
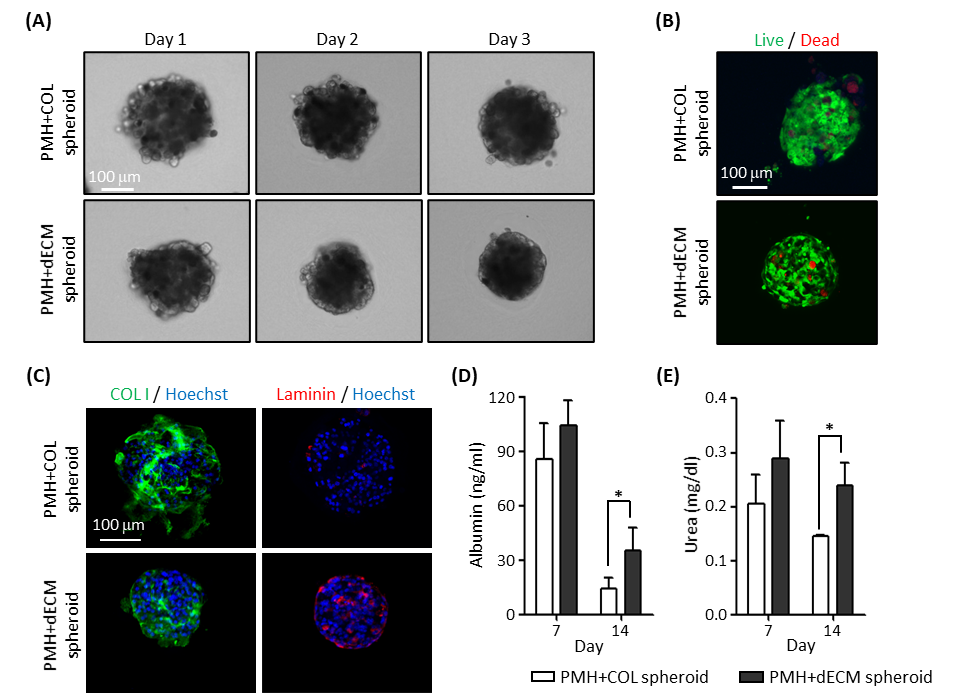
**

**Supplementary Figure 5. Bioprinted, collagen- and dECM-incorporated PMH spheroids and their hepatic functions.** Optical microscope images (A) and live/dead staining results (B) of collagen- and dECM-incorporated PMH spheroids. The live/dead staining was conducted on day 3 after printing. (C) Immunostaining results of the spheroids on day 3. Measured albumin (D) and urea (E) secretions of the spheroids on day 7 and 14 (n=5, *p < 0.05).
